# Morphological Evolution of TiB_2_ and TiAl_3_ in Al–Ti–B Master Alloy Using Different Ti Adding Routes

**DOI:** 10.3390/ma15061984

**Published:** 2022-03-08

**Authors:** Yanjun Zhao, Zepeng Lu, Li Mi, Zhiliu Hu, Wenchao Yang

**Affiliations:** 1 College of Resources, Environment and Materials, Guangxi University, Nanning 530004, China; 1915391021@st.gxu.edu.cn (Z.L.); huzhiliu@gxu.edu.cn (Z.H.); ywch053@163.com (W.Y.); 2 Guangxi Key Laboratory of Processing for Non-ferrous Metal and Featured Materials, Guangxi University, Nanning 530004, China; 3 AECC South Industry Co., Ltd., Zhuzhou 412002, China; milo12271@163.com

**Keywords:** Al–Ti–B Master Alloy, TiB_2_, TiAl_3_, halide salt route, “Ti-sponge” route, “partial Ti-sponge” route, Ti–TiAl_x_ mechanism

## Abstract

Three different Ti addition routes were used to prepare an Al–5Ti–B Master Alloy: the halide salt route, the Ti-sponge route, and the partial Ti-sponge route. In the halide salt route, the raw materials were Al + KBF_4_ + K_2_TiF_6_; K_2_TiF_6_ was completely replaced by pure titanium for the Ti-sponge route versus the halide salt route; in the partial Ti-sponge route, K_2_TiF_6_ was partially replaced by pure titanium. Here, 30% Ti-sponge or 60% Ti-sponge route means that 30% or 60% K_2_TiF_6_ was replaced by pure titanium, respectively. The above Ti addition routes have a significant influence on the growth pattern and morphological evolution of TiAl_3_ and TiB_2_, which greatly affect the refining performance of Al–Ti–B Master Alloy. When using the halide salt route, a streamlined “rich Ti, B area” exists in the aluminum melt, which is a complex compound of (Ti_x_, Al_1−x_) B_y_. The “rich Ti, B area” is essential for the nucleation and growth of TiAl_3_ and TiB_2_. Blocky TiAl_3_ was obtained and its average size was 4.7 μm based on the halide salt route. In the Ti-sponge route, the nucleation of TiAl_3_ mainly depends on the mutual diffusion of Al and Ti, and TiAl_x_ forms around pure Ti particles, i.e., the so-called Ti–TiAl_x_ mechanism. The average size of the blocky TiAl_3_ was 9.8 μm based on the Ti–TiAl_x_ mechanism. For the partial Ti-sponge route, the “rich Ti, B area” gradually decreases with the increase in Ti powder’s contents, and large TiAl_3_ coexists with the small TiAl_3_. Compared with the Ti-sponge route, the halide salt route can form smaller TiAl_3_. In the Ti-sponge route, there is a small amount of “rich Ti, B area” due to the influence of the Ti–TiAl_x_ mechanism, which does not meet the requirements of TiB_2_ growth. In the halide salt route, there is sufficient “rich Ti, B area”, which is conducive to the formation of TiB_2_. Both the crystal defects and the crowded growth environment caused by the “rich Ti, B area” are fundamental reasons for the fragility and the irregular shape of the TiB_2_. The refining effect of the Al–Ti–B Master Alloy prepared by the halide salt route is better than the Ti-sponge route. The refining effect of 30% Ti-sponge route is better than that of Ti-sponge route and worse than that of halide salt route.

## 1. Introduction

Al–Ti–B Master Alloy is an essential grain refiner and can strongly refine the microstructure and prevent the formation of coarse and equiaxed grains as well as columnar grains during the casting process of aluminum and aluminum alloy. It can also make the ingot’s microstructure become uniform, reduce segregation, and inhibit cracks. This greatly improves the mechanical and physical properties of aluminum and aluminum alloys [1,2,3,4,5]. The refinement mechanisms of Al–Ti–B Master Alloy are complicated and mainly include carbide–boride particle theory [6], peritectic reaction theory [7], peritectic hulk theory [8], and the duplex nucleation mechanism [9]. Of these, the duplex nucleation theory has attracted extensive attention [10]. The theory shows that after the Al–Ti–B was added into the aluminum melt, the melting of TiAl_3_ releases Ti. Ti then segregates toward TiB_2_ surface, which leads to an increase in the Ti concentration around TiB_2_ and formation of TiAl_3_ outside of TiB_2_. In this theory, the TiB_2_ phase plays an indirect nucleation role. The duplex nucleation mechanism indicates that a smaller size of TiB_2_ and TiAl_3_ leads to a more uniform distribution of the second phase—this in turn leads to better refining of the Al–Ti–B Master Alloy.

The preparation methods of Al–Ti–B mainly include the halide salt route [11,12], the Ti-sponge route [13], the self-propagating high-temperature synthesis method [14,15], and the reprecipitated TiB_2_ particles method [16]. The halide salt route and the Ti-sponge route are the primary methods for preparing. The halide salt route adds K_2_TiF_6_ and KBF_4_ into the aluminum melt according to the Ti/B value of the required product. The related reactions during the halide salt route are shown in Formulas (1)–(3) [17]. It is low-cost, simple production equipment and offers continuous production of stable and efficient refining effects for Al–Ti–B [13,18]. The Ti-sponge route replaces the K_2_TiF_6_ in the halide salt route with Ti powder, as shown in Formulas (4) and (5) [19]. The formation and evolution mechanisms of TiAl_3_ and TiB_2_ are different for the halide salt route and the Ti-sponge route.

(1) $$6{\mathrm{KBF}}_{4}\left(l\right)+3K_{2}{\mathrm{TiF}}_{6}\left(l\right)+10\mathrm{Al}\left(l\right)\to 3{\mathrm{TiB}}_{2}\left(s\right)+9{\mathrm{KAlF}}_{4}\left(l\right)+K_{3}{\mathrm{AlF}}_{6}\left(l\right)$$

(2) $$3K_{2}{\mathrm{TiF}}_{6}\left(l\right)+13\mathrm{Al}\left(l\right)\to 3{\mathrm{TiAl}}_{3}\left(s\right)+3{\mathrm{KAlF}}_{4}\left(l\right)+K_{3}{\mathrm{AlF}}_{6}\left(l\right)$$

(3) $$2{\mathrm{KBF}}_{4}\left(l\right)+3\mathrm{Al}\left(l\right)\to {\mathrm{AlB}}_{2}\left(s\right)+2{\mathrm{KAlF}}_{4}\left(l\right)$$

(4) $$2{\mathrm{KBF}}_{4}\left(l\right)+2\mathrm{Ti}+5\mathrm{Al}\left(l\right)\to {\mathrm{TiB}}_{2}\left(s\right)+{\mathrm{TiAl}}_{3}\left(s\right)+2{\mathrm{KAlF}}_{4}\left(l\right)$$

(5) $$2{\mathrm{KBF}}_{4}\left(l\right)+3\mathrm{Al}\left(l\right)\to {\mathrm{AlB}}_{2}\left(s\right)+2{\mathrm{KAlF}}_{4}\left(l\right)$$

Liu [20] studied the relationship between the morphology of TiAl_3_ and the smelting temperature for the halide salt route. Low-temperature smelting can form block-shaped TiAl_3_, while high-temperature smelting can quickly form rod-shaped TiAl_3_ crystals. Xie [21] also found that TiAl_3_ mainly has two morphologies: block-shaped and rod-shaped; the solute Ti grows easily around the agglomerate TiAl_3_, and TiB_2_ also has specific morphological evolution. TiB_2_ particles are hexagonal and independent when Al–Ti–B Master Alloy was prepared by the halide salt route [22]. The reaction temperature does not influence the morphology of TiB_2_. However, TiB_2_ particles showed different morphologies at different reaction temperatures when the Master Alloy was prepared by the Ti-sponge route [12]. The TiB_2_ particles are larger than 5 μm when the reaction temperature is 850 °C. When the reaction temperature reached 1200 °C, TiB_2_ particles gradually changed into layered stacking morphology and even a dendritic morphology. Zhang [23] confirmed by kinetic analysis that TiB_2_ particles are not stable in Al melt and Ti addition can suppress the dissolution of TiB_2_ particles. TiB_2_ may coarsen during the holding temperature and grow during the cooling of the melt. Wang [24] demonstrates a strong epitaxial growth of TiAl_3_ on the surface of TiB_2_ particles. Fan et al. also found that a layer of TiAl_3_ was formed on the surface of TiB_2_ and that TiAl_3_ can significantly improve the stability of TiB_2_ [25]. TiAl_3_ and TiB_2_ also interact with each other. Wang et al. also proved that TiB_2_ is critical to grain refinement of aluminum and aluminum alloys [26]. TiB_2_ particles react with aluminum slowly and release Ti into the melt. The TiAl_3_ particles then combine with Ti in the melt to form a dynamic Ti-rich layer on the surface of (Ti, Al) B_2_. This layer offers a low crystal mismatch with α-Al and promotes the nucleation of aluminum grains.

The Ti-sponge route replaces K_2_TiF_6_ in the halide salt route with Ti powder. According to the addition amount of raw materials, the amount of K_2_TiF_6_ needed, for the same content of Ti, is five times that of Ti powder. The preparation of 1 ton of Al–5Ti–B master alloy needs 0.3665 tons of mixed fluoride salt of K_2_TiF_6_ and KBF_4_ but only 0.1665 tons of Ti powder and KBF_4_. The liquid salt slags and fluoride gas produced by Ti-sponge route are much lower than that of the halide salt route. Therefore, the fluoride pollution of halide salt route is much more serious than that of the Ti-sponge route. The partial Ti-sponge route of combining the halide salt route and the Ti-sponge route may have a better refining effect and improved environmental effects. It is necessary to explore the refining effect of Al–Ti–B Master Alloy using different Ti adding routes.

The growth pattern and morphology distribution of the TiAl_3_ and TiB_2_ for different preparation processes have not been thoroughly studied. The difference between the Ti-sponge route and halide salt route is quite rarely analyzed. Here, an Al–Ti–B Master Alloy was prepared using different Ti addition routes including the halide salt route, the Ti-sponge route, and the partial Ti-sponge route. The growth pattern and morphological evolution of the TiAl_3_ and TiB_2_ were analyzed, and the refining effects using different Ti adding routes were also discussed.

## 2. Experimental

Table 1 shows the experimental parameters of Al–5Ti–B Master Alloy prepared by the halide salt route. Pure aluminum, KBF_4_, and K_2_TiF_6_ were all added at a nominal composition of the Al–5Ti–B Master Alloy (Al:Ti:B = 100:12.4:26.6, weight ratio). The dried pure aluminum was heated to 800 °C in a graphite crucible until the metal was soft-crushed. A coating agent (10.8% calcium fluoride + 72.8% magnesium chloride + 16.4% chlorinated calcium) was then covered on the surface of the aluminum melt. The slag was removed after a hexachloroethane refiner was added at 720 °C and held for 1200 S. When increasing temperature to the experimental reaction temperature (Table 1), mixed fluoride salt (KBF_4_ and K_2_TiF_6_ were premixed before adding) was added into the aluminum melt, stirred evenly, and held for 10–1800 S (Table 1). The melt was then poured into a preheated steel mold to obtain a cubic ingot (160 × 100 × 12 mm).

Table 2 shows the experimental parameters of Al–5Ti–B Master Alloy prepared by the partial Ti-sponge route and Ti-sponge route. Based on the halide salt route, K_2_TiF_6_ was gradually replaced by pure Ti. The Ti-sponge route means that K_2_TiF_6_ was totally replaced by pure Ti. The 30% Ti-sponge route and 60% Ti-sponge route mean that K_2_TiF_6_ was partially replaced by 30% Ti and 60% Ti, respectively.

The microstructure samples were cut from the center of the ingots at the center height, mounted, and mechanically polished. The samples were then etched using Keller’s reagent for 10 s. The microstructures were characterized using a Leica DMR research optical microscope (OM) and a SU-8020 field emission gun scanning electron microscope (FEG SEM) operating at 45 kV and a tube current of 40 μA. A D/max 2500V X-ray diffractometer equipped with Ni-filtered Cu K_α_ radiation source scanning over a range of 2θ = 20–80° was used to analyze TiB_2_ and TiAl_3_.

## 3. Results

### 3.1. “Rich Ti, B Area” and Morphology of TiAl_3_, TiB_2_ in Halide Salt Route

**Figure 1:**
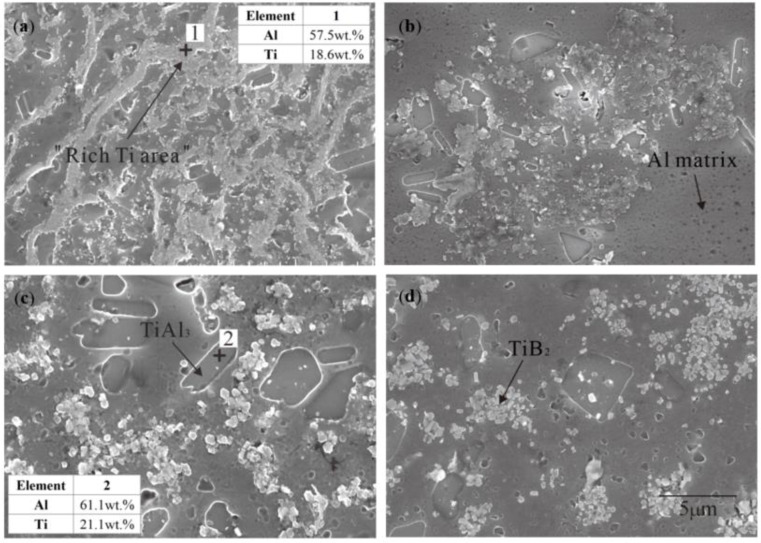
Microstructure and EDS of Al–Ti–B Master Alloy prepared by the halide salt route with different reaction times at 800 °C: (**a**) 10 S; (**b**) 30 S; (**c**) 60 S; (**d**) 600 S.

The possible reactions during the Al–Ti–B Master Alloys prepared by the halide salt route are shown in Formulas (1)–(3). These reactions are the main sources of TiAl_3_ and TiB_2_. Figure 1 shows the microstructure and composition of Al–Ti–B prepared by the halide salt route at 800 °C with different reaction times. There are obvious filamentous areas in Figure 1a. According to the EDS analysis, the filamentous areas were rich in elemental Ti but did not contain element F. This indicates that these areas should be the product areas of the fluoride salt reaction. The ratio of Ti to Al at point 2 is about 34.5%, which is nearly equal to the mass fraction of Ti in TiAl_3_ (about 36.96%). Considering that Al may be oxidized, point 2 should be TiAl_3_. Point 1 contains Ti and the energy spectrometer cannot detect the existence of B; therefore, we infer that the filamentous area is at least rich in Ti. The filamentous area gradually disappeared with longer reaction times. The filamentous area greatly reduced at 30 S (Figure 1b), and TiAl_3_ of thin rods or small blocks and aggregative TiB_2_ appeared. The filamentous area disappeared at 60 S (Figure 1c). At this point, the TiAl_3_ became thick rods and large blocks, and TiB_2_ dispersed into coarse grains. When the reaction time was extended to 600 s, the TiB_2_ further dispersed into fine particles (Figure 1d), thus distributing in the Al matrix. TiB_2_ was generated in the incipient filamentous area, which suggests that the filamentous Ti-rich area was also rich in B. Therefore, the filamentous area should be called the “rich Ti, B area”.

**Figure 2:**
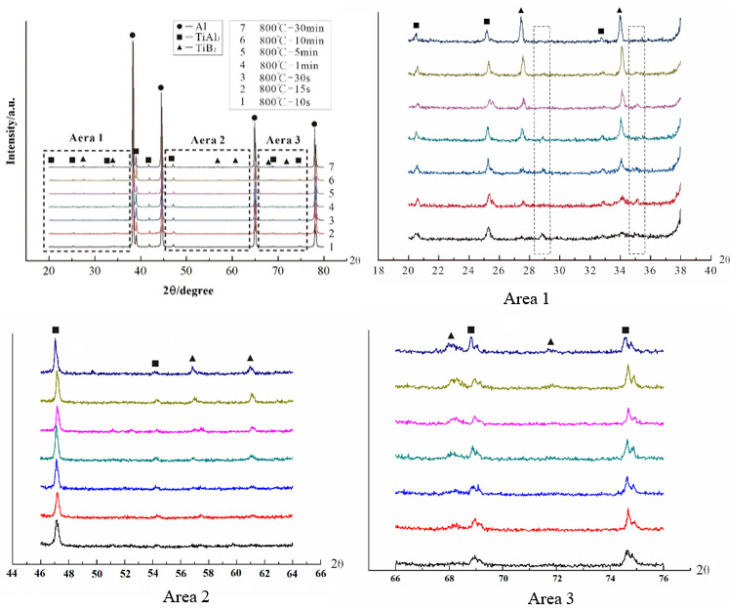
XRD pattern of Al–Ti–B Master Alloy prepared by the halide salt route at 800 °C for different reaction times.

The XRD spectra at 800 °C for different reaction times are shown in Figure 2 to further confirm the “rich Ti, B area”. The main phases of all samples were Al, TiAl_3_, and TiB_2_, and no prominent excess peaks were observed. The intensity of the TiAl_3_ peak is basically the same from 10 S to 1800 S, which indicates that TiAl_3_ generated at the initial stage of the halide salt reaction. The TiB_2_ peak obviously changed from weak to strong. There is no TiB_2_ peak in the XRD pattern at 10 S and a weak TiB_2_ peak appears at 15 S, thus indicating that the formation of TiB_2_ is slower than that of TiAl_3_ or it generates too little to detect in the early stage. The TiB_2_ and AlB_2_ have a similar crystal structure, and thus, diffraction peaks may be broadened or separated in XRD diffraction. If the value of Ti/B is greater than 2.2, then excess B will form AlB_2_ [27]. Therefore, the peaks in the two square frames in Area1 were considered to be AlB_2_, which are very close to the strong peak of TiB_2_. A small amount of AlB_2_ also exists in the early stage of the reaction. Some studies have reported the nonhomogenous distribution of solutes B and Ti close to the salts/Al melt interface during the reaction of the fluoride salts and Al melt. These form unstable AlB_2_. The AlB_2_ transforms to TiB_2_ via diffusion of solutes Ti and B in the aluminum melt in the subsequent holding temperature process [28,29]. The essence of filamentous areas (Figure 1a) is a complex compound (Ti_x_, Al_1−x_)B_y_; this area was called the “rich Ti, B area”. In the early stage of the halide salt reaction, the streamlined “rich Ti, B area” existed in the aluminum melt (Figure 1a). The formation of TiAl_3_ was very fast (less than 10 S). The “rich Ti, B area” was the birthplace of the nucleation and growth of initial TiAl_3_ and TiB_2_ for the halide salt route.

**Figure 3:**
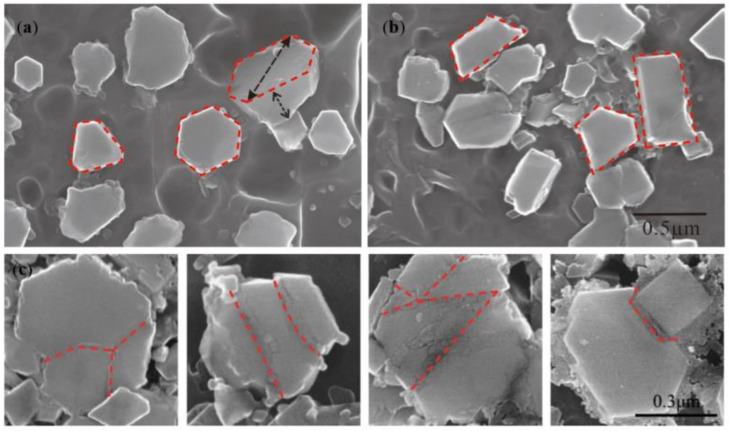
Morphology of TiB_2_ in the Al–Ti–B Master Alloy prepared by the halide salt route at 800 °C for 600 s: (**a**) hexagonal TiB_2_; (**b**) irregular polygon TiB_2_; (**c**) mutual ”crashing” of fragile TiB_2_.

Figure 3 clearly shows the morphology of the TiB_2_ in the Al–Ti–B Master Alloy prepared by the halide salt route at 800 °C for 300 S. The white shadow on the particle’s edge shows that TiB_2_ is hexagonal with a certain thickness. The maximum section size of these hexagons is 0.5–1.0 μm. In the statistics of T.E. Quested [28], the size of TiB_2_ particles in conventional Al–Ti–B Master Alloy is about 5 μm, and the size of most TiB_2_ is 0.5–1.5 μm. Figure 3b shows the irregular TiB_2_; they may be the side cuts of the hexagonal TiB_2_; however, the mutual “crashing” during fragile TiB_2_′s growth also may lead to the forming of the irregular shape (Figure 3c).

**Figure 4:**
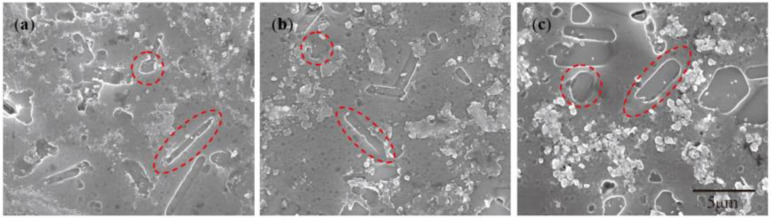
Morphology of TiAl_3_ in the Al–Ti–B Master Alloy prepared by the halide salt route after different reaction times at 800 °C: (**a**) 15 S; (**b**) 30 S; (**c**) 60 S.

Figure 4 shows the morphology of TiAl_3_ prepared by the halide salt route at 800 °C for 15 S, 30 S, or 60 S. At 800 °C for 15 S, the TiAl_3_ was granular or elongated rod-shaped. When the reaction time was extended to 30 S, the strip phase became a thick rod. TiAl_3_ was blocky when the reaction time was 60 S.

### 3.2. Morphology of TiAl_3_, TiB_2_ in the Ti-Sponge and Partial Ti-Sponge Route

**Figure 5:**
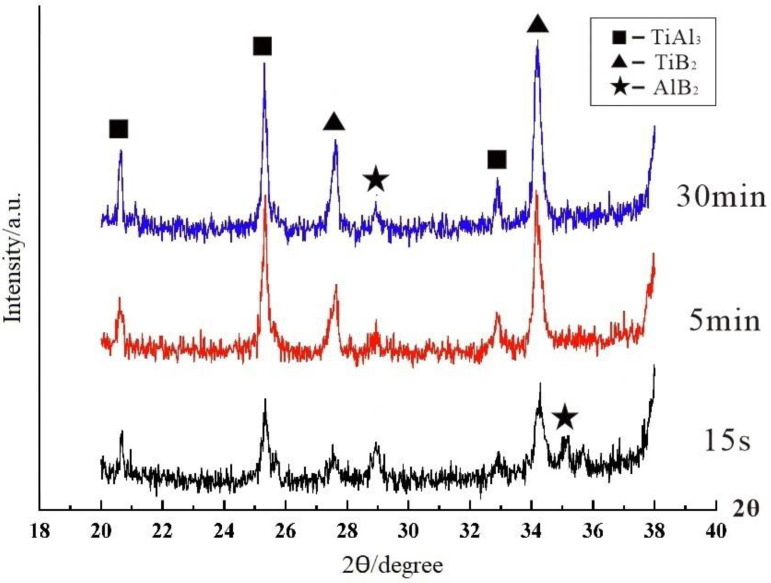
XRD analysis of Al–Ti–B Master Alloy prepared by Ti-sponge route at 800 °C with different reaction times.

The reactions of preparing Al–Ti–B Master Alloy by the Ti-sponge route are shown in Formulas (4) and (5). Reaction (4) occurred spontaneously at the temperature range of 800–900 °C [19]. The mass ratio of Ti to B is 4.43:1 in reaction (4). The mass ratio of Ti/B added to the reaction is 5:1. Therefore, reaction (5) also occurred. Figure 5 shows the X ray diffraction pattern of the Al–Ti–B Master Alloy prepared by the Ti-sponge route with reaction times of 15 S, 300 S, and 1800 S. The diffraction peaks of the TiB_2_ were observed at 15 S. AlB_2_ and TiB_2_ existed in the matrix simultaneously at 15 S (consistent with Formula (5)) for TiB_2_ diffraction peaks broadening.

**Figure 6:**
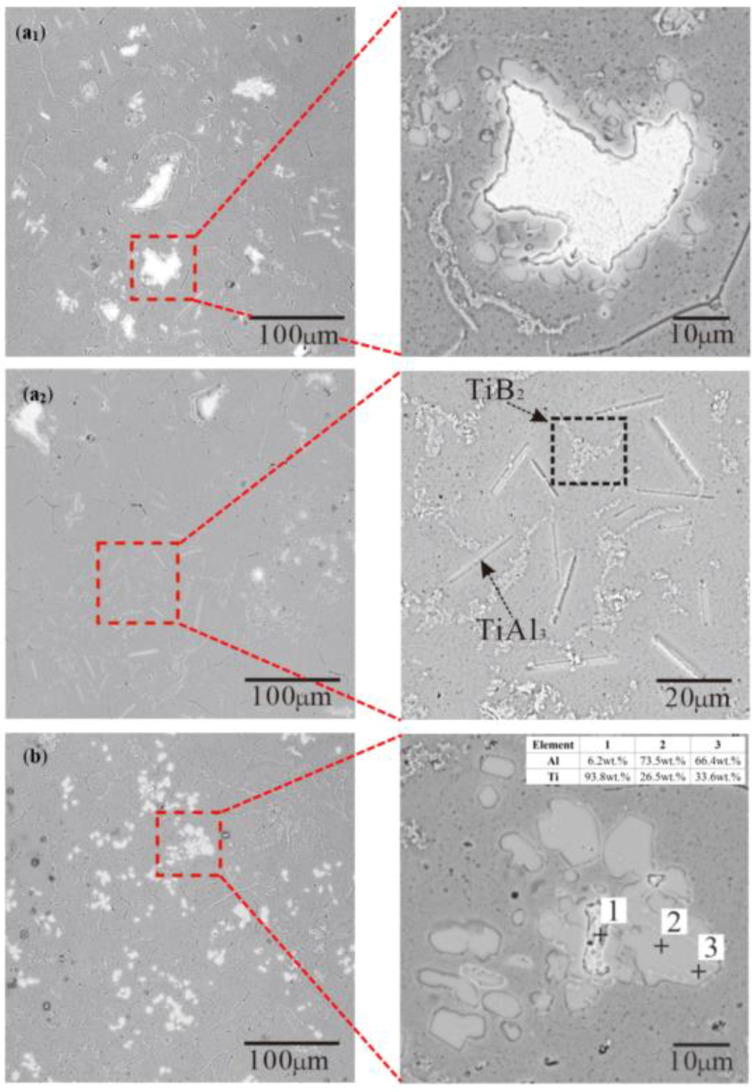
The microstructure and EDS of Al–Ti–B Master Alloy prepared by the Ti-sponge route at 800 °C: (**a_1_**) 15 S reaction time—irregular blocky TiAl_3_; (**a_2_**) 15 S reaction time—fine, rod-shaped TiAl_3_; (**b**) 300 S reaction time.

**Figure 7:**
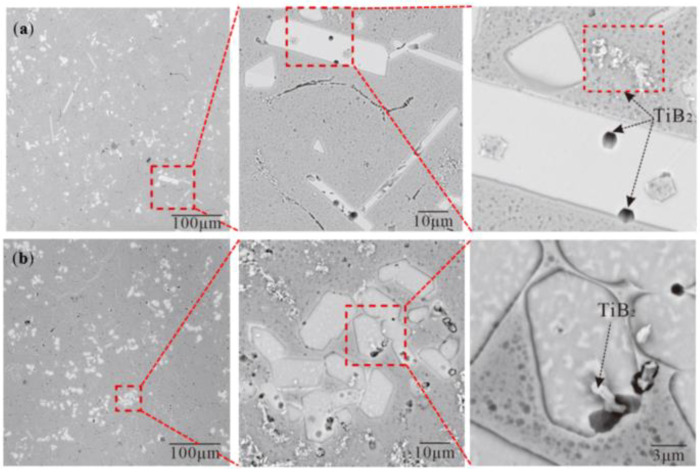
Microstructure of Al–Ti–B Master Alloy prepared by partial Ti-sponge route at 800 for 300 S: (**a**) 30% Ti-sponge route; (**b**) 60% Ti-sponge route.

The microstructure and EDS of Al–Ti–B Master Alloys prepared by the Ti-sponge route and partial Ti-sponge route at 800 °C for different times are shown in Figure 6 and Figure 7. Figure 6a_1_,a_2_ shows different parts of the same sample. Owing to the short reaction time of 15 S, some Ti powders did not have enough time to diffuse. Therefore, the irregular white blocky TiAl_3_ was observed in Figure 6a_1_. Other Ti powders are relatively dispersed to form fine, rod-shaped TiAl_3_ (Figure 6a_2_). At a reaction time of 300 S, the whole block dispersed into small, irregular pieces (Figure 6b). The original fine, rod-shaped structure disappeared. The energy spectrum analysis indicated the white phase in Figure 6b should be pure Ti, and the surrounding gray phase should be TiAl_x_. The concentration of Al in the gray phase decreased gradually from inside to outside while Ti increased. The value of Ti/Al at point 3 was closer to 36.96%, which is TiAl_3_.

**Figure 8:**
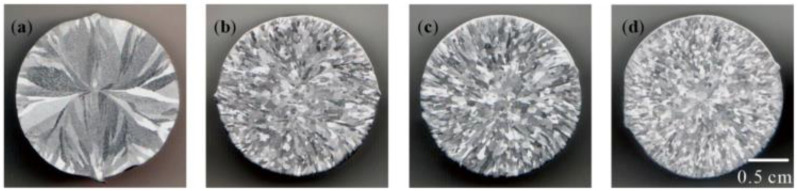
The refining effect of different Al–Ti–B Master Alloy on pure aluminum: (**a**) Pure aluminum; (**b**) 30% Ti-sponge route; (**c**) Ti-sponge route; (**d**) halide salt route.

There are both blocky and rod-shaped TiAl_3_ in Figure 8. Further, it was found that TiB_2_ was embedded in blocky TiAl_3_ (Figure 7b). The hexagonal holes appeared in the rod-shaped TiAl_3_ and were eroded by pure Al (Figure 7a). If B was added into the Al–Ti alloy melt containing TiAl_3_, then B could form TiB_2_ on the TiAl_3_ and cause the TiAl_3_ to break or even dissolve [29]. The resulting TiB_2_ will be agglomerated between the TiAl_3_ and Al matrix. The reaction equation is as follows:

(6) $${\mathrm{TiAl}}_{3}\left(s\right)+2B\to {\mathrm{TiB}}_{2}\left(s\right)+3\mathrm{Al}\left(l\right)$$

### 3.3. Refining Effect of Al–Ti–B Master Alloy

The Al–Ti–B Master Alloy obtained at 800 °C for 1800 S using different routes was used to refine pure aluminum ingot; the refined microstructure is shown in Figure 8. The average grain sizes of pure aluminum ingot refined by halide salt route, 30% Ti-sponge route, and Ti-sponge route are 98.3 μm, 105.4 μm, and 111.8 μm, respectively. The refining effect of 30% Ti-sponge route is better than that of Ti-sponge route and worse than that of halide salt route.

The size of TiAl_3_ obtained using the Ti-sponge route was larger than that using the halide salt route; they are 9.83 μm (Figure 6) and 4.7 μm (Figure 4), respectively. In the 30% Ti-sponge or 60% Ti-sponge route, the large-sized granular TiAl_3_ was coexistent with the small-sized TiAl_3_ (Figure 4). The size of TiB_2_ particles is about 5 μm, and the size of most TiB_2_ is 0.5–1.5 μm (Figure 3). The refining effect of Al–Ti–B Master Alloy is related to the size and distribution of TiAl_3_ and TiB_2_. A finer size and more uniform distribution of TiAl_3_ and TiB_2_ causes the Al–Ti–B Master Alloy to have a better grain-refining effect.

## 4. Discussion

### 4.1. Nucleation and Growth of TiAl_3_ and TiB_2_ in Different Ti Adding Routes—The Ti–TiAl_x_ Mechanism

**Figure 9:**
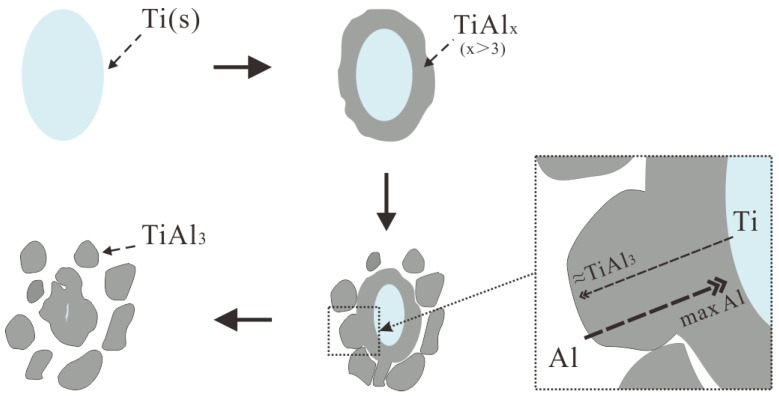
The Ti–TiAl_x_ mechanism in Ti-sponge route to prepare Al–Ti–B Master Alloy.

The nucleation of TiAl_3_ mainly depends on the mutual diffusion of Al and Ti for the Ti-sponge route. This diffusion process makes TiAl_x_ form around the pure Ti particles first, as shown in Figure 6b. As diffusion progresses, the external TiAl_x_ is separated into single TiAl_3_, but the concentration of internal Ti remains high (Figure 6a_1_). Finally, the TiAl_x_ completely converted into separated TiAl_3_ after 300 S reaction at 800 °C (Figure 6b). This mechanism is called the Ti–TiAl_x_ mechanism for the Ti-sponge route, as shown in Figure 9. Sunda [30] proposed that Ti–Al’s interdiffusion coefficient (D) is a function of composition and temperature; D increases with increasing temperature when the composition is constant. The diffusion activation energy of aluminum (Q) is different in different phases, but Q is substantially constant and independent of the concentration. We have the following order: Q[TiAl_3_] > Q[TiAl] > Q[α-Ti(Al)] > Q[β-Ti(Al)]. Obviously, the diffusion coefficient of Al in Ti3Al is smaller than that in Ti particles. Therefore, Al enriched on the Ti particle’s surface passed through the TiAl_3_ layer (Figure 9) resulting in a high concentration of Al surrounding the surface of the Ti particles. Thus, TiAl_x_ is constantly formed and eventually converted into separate TiAl_3_.

The nucleation mechanism of the rod-shaped TiAl_3_ (Figure 6a_2_) is different from the Ti–TiAl_x_ mechanism. The atomic ratio of Ti to KBF_4_ is 1:1 in reaction (4). Actually, the ratio of Ti to KBF_4_ is about 1.13:1 in this experiment so that KBF_4_ can react completely. The rod-shaped TiAl_3_ comes from reaction (4) and is easy to dissolve versus blocky TiAl_3_; therefore, it almost disappeared after 300 S of reaction (4), as shown in Figure 6b. For the Ti-sponge route, Ti is involved in the transition of Ti–TiAl_x_ to produce TiAl_3_, which leads to the deficiency of Ti and excess of B. The B can form TiB_2_ on the TiAl_3_ and cause TiAl_3_ to break and be dissolved. The long, rod-shaped TiAl_3_ is more easily dissolved than the block-shaped TiAl_3_ [31]. Therefore, in the Ti-sponge route, the initial formation of the rod-shaped TiAl_3_ does not stay easily in the melt, which is different from the characteristic of the halide salt route. However, there is still a rod-shaped TiAl_3_ in the microstructure of the Al–Ti–B Master Alloy prepared by the 30% Ti-sponge route with the reaction temperature of 800 °C for 900 S. The total amount of Ti is constant, and with less free Ti content, there is less of a Ti–TiAl_x_ mechanism. When there is less excess B, some of the rod-shaped TiAl_3_ are retained and less Ti is consumed in the melt.

**Figure 10:**
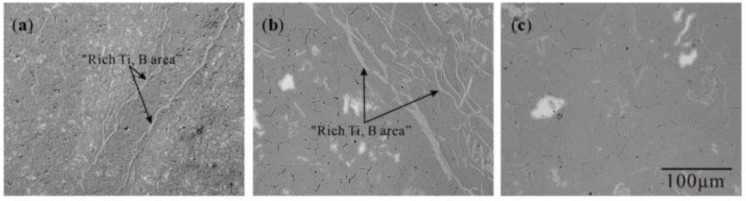
The “rich Ti, B area” in different Al–Ti–B Master Alloy prepared routes at 800 °C for 15 S: (**a**) halide salt route; (**b**) 60% Ti-sponge route; (**c**) Ti-sponge route.

Section 3.1 shows that the “rich Ti, B area” is the birthplace of the nucleation and growth of initial TiAl_3_ and TiB_2_ for the halide salt route. Similarly, the “rich Ti, B area” are also found in the partial Ti-sponge route as shown in Figure 10b. The “rich Ti, B area” gradually decreased with increasing Ti powder content; only a small amount of filamentous and small strip regions were formed in the Ti-sponge route. In Ti-sponge route, a lot of Ti powder is directly involved in the formation of TiAl_3_ by the Ti–TiAl_x_ mechanism. During the nucleation and growth of TiAl_3_, the blocky Ti powder seriously hindered the diffusion of Ti into the Al melt, thus resulting in insufficient Ti in the melt. The insufficient diffusion of Ti also severely reduced the amount of Ti involved in the reaction (4), thus reducing the “rich Ti, B area” in the melt.

The Ti addition routes have greatly influenced the morphology and size of TiAl_3_. The size of TiAl_3_ formed based on the Ti–TiAl_x_ mechanism in the Ti-sponge route is coarse. Ti and Al diffused mutually in the TiAl_x_ around the Ti particles, and the outer TiAl_3_ is mainly separated into small blocks. The “rich Ti, B area” in the Ti-sponge route under the influence of the Ti–TiAl_x_ mechanism is a small amount that cannot meet the TiB_2_ growth well. Due to the Ti–TiAl_x_ mechanism, the diffusion efficiency of Ti in Ti-sponge route is much lower than halide salt route. TiAl_3_ formed based on the chemical reaction (4) is long and rod-shaped. The long, rod-shaped TiAl_3_ cannot exist stably in the melt due to the excess B and lack of Ti in the pre-reaction melt. Therefore, it does not appear in the final microstructure.

### 4.2. Evolution Mechanism of TiB_2_ with Irregular Polygon

In addition to reaction (1), the following reaction may produce TiB_2_ when the Al–Ti–B Master Alloy is prepared by the halide salt route [32]:

(7) $${\mathrm{AlB}}_{2}\left(s\right)+\mathrm{Ti}\to {\mathrm{TiB}}_{2}\left(s\right)+\mathrm{Al}\left(l\right)$$

(8) $$2B+\mathrm{Ti}\to {\mathrm{TiB}}_{2}\left(s\right)$$

**Figure 11:**
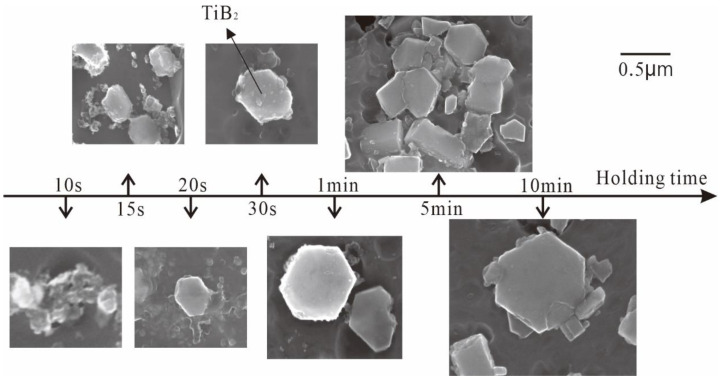
Morphology of TiB_2_ for the halide salt route at 800 °C for different reaction times.

Here, TiB_2_ is nucleated by the fluorination reaction and grown in the rich Ti and B area based on the above two reactions. Figure 11 shows the microstructure of the TiB_2_ when the mixed fluoride salt is added into the aluminum melt at 800 °C for 10 S–600 S using the halide salt route.

The TiB_2_ was an irregular small particle at the initial stage of the reaction (10 S and 15 S). Regular hexagonal TiB_2_ particles appeared with longer reaction times to 20 S and 30 S, and the size of TiB_2_ was about 0.5 μm. At 60 S, the size of TiB_2_ particle continued to increase. At 5 min, some TiB_2_ collided together and cracks appeared along the junction of particles. At a reaction time of 10 min, the size of TiB_2_ particles reached 1.5 μm and there were a lot of lug boss’ around the TiB_2_ particles. According to the reaction (7), the filamentous AlB_2_ (corresponding to (Ti_x_, Al_1−x_)B_y_ in Figure 1a) was more likely to be destroyed when it was transformed into TiB_2_ [33]. Therefore, TiB_2_ was more easily crushed with increasing reaction time.

**Figure 12:**
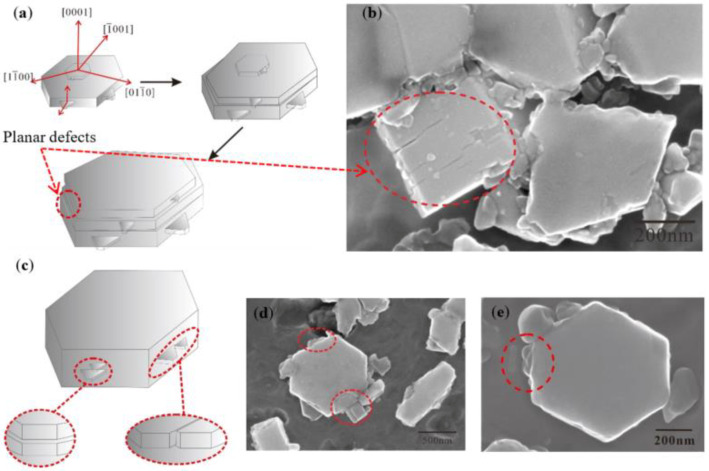
Schematic diagram and microstructure of possible defects in the earlier and later growth stage of TiB_2_ for the Al–Ti–B Master Alloy prepared by the halide salt route: (**a**) earlier TiB_2_ crystal growth model; (**b**) earlier actual growth of TiB_2_; (**c**) later TiB_2_ crystal growth model; (**d**,**e**) later actual growth of TiB_2_.

The crushing of TiB_2_ is related to the crystal structure. Figure 12a,c represent the crystal growth model of TiB_2_ [34]. TiB_2_ belongs to hexagonal system (the lattice parameters a = 0.3030 nm, c = 0.3232 nm, c/a = 1.0818). The growth direction extends longitudinally in the direction of <0001> or extends laterally in the direction of <1$\bar{1}$00>. The growth rate is the slowest because the {0001} surface has the highest atomic density and the lowest surface energy. Thus, the longitudinal growth rate of TiB_2_ is lower than the lateral expansion speed. The lug boss in <0001> direction and the projecting lug in <1$\bar{1}$00> direction were also observed, as shown in Figure 12b,d,e.

In the nucleation of TiB_2_, the generation and expansion mechanism of lug boss plays a significant role. The contents of Ti and B in the “rich Ti, B area” are sufficient, and the TiB_2_ (0001) surface is small and provides favorable conditions for the formation of lug boss. When the lug boss is generated in the [1] direction, the lug boss rapidly expands the entire (0001) plane, and the thickness and size of the TiB_2_ quickly increase. The nucleation of TiB_2_ is similar to the layered stacking pattern according to the above mechanism (Figure 12a,b). There may be parallel surface defects between the new layer formed by the lug boss and the original layer. The formation and expansion mechanism of the projecting lug play a significant role during the TiB_2_ growth. The Ti and B in the “rich Ti, B area” were partially consumed at TiB_2_ growth stage. The growth of the {0001} plane requires larger surface energy, which makes the formation and expansion of the lug boss difficult. Therefore, the thickness of the TiB_2_ does not change. The growth of TiB_2_ is the lug generation and expansion in the direction <1$\bar{1}$00>.

Figure 12c–e show that many lugs in the {1100} plane are prone to producing crystal defects during simultaneous growth. When the two lugs grow in parallel, there may be a planar defect parallel to the {1100} plane between the upper and lower lugs. When the two lugs grow side by side, between the left and right lugs, it is possible to form a planar defect perpendicular to the {1100} plane. The {1100} plane is not flat when the lug stops the growth eventually due to the lack of Ti and B.

**Figure 13:**
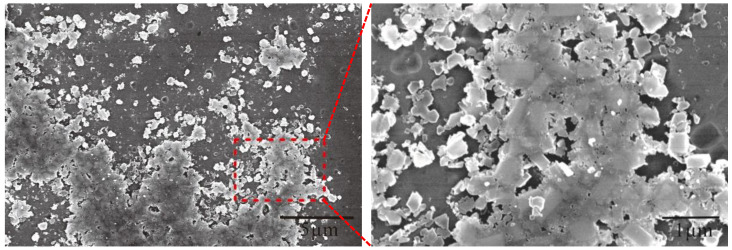
SEM of the “rich Ti, B area” and TiB_2_ in the Al–Ti–B Master Alloy prepared by the halide salt route at 800 °C for 60 S.

The crushing of the TiB_2_ is also related to the growth environment. Figure 13 shows the microstructure of the “rich Ti, B area” for the halide salt route at 800 °C for 60 S. The TiB_2_ was rich in “rich Ti, B area” and many TiB_2_ particles were clustered together during the growth process. This aggregation phenomenon can also crush TiB_2_. It is difficult to ensure uniform growth in all directions; thus, TiB_2_ has an irregular morphology.

## 5. Conclusions

(1) The fluorine salt reaction occurred very fast when Al–Ti–B was prepared by the halide salt route (<10 S). A streamlined “rich Ti, B area” exists in the aluminum melt in the initial stage of the reaction ((800 °C, <30 S). This is a complex compound of (Ti_x_, Al_1−x_) B_y_. The “rich Ti, B area” is essential for the nucleation and growth of TiAl_3_ and TiB_2_.
(2) The Ti addition route greatly influences the morphology of TiAl_3_. The formation of TiAl_3_ using the Ti-sponge route is based on the Ti–TiAl_x_ mechanism. The nucleation of TiAl_3_ mainly depends on the mutual diffusion of Al and Ti powder, and TiAl_x_ formed around the Ti particles. The TiAl_3_ formed based on the Ti–TiAl_x_ mechanism is mainly block-shaped and the average size is 9.83 μm. In the halide salt route, TiAl_3_ formation is based on the reaction of Ti powder and KBF_4_ to form a rod-shaped TiAl_3_. The long, rod-shaped TiAl_3_ disappeared due to excess B and a lack of Ti in the aluminum melt. Finally, small blocks of TiAl_3_ formed with an average size of 4.7 μm.
(3) TiB_2_ particles showed different morphologies at different reaction times when the Master Alloy was prepared by the halide salt route. The TiB_2_ was an irregular and small particle at the initial stage of the reaction (10 S and 15 S). Regular hexagonal TiB_2_ particles appeared as the reaction time increased to 20 S and 30 S. The size of TiB_2_ was about 0.5 μm. The size of TiB_2_ particle continued to increase at 60 S. Some TiB_2_ collided together at 5 min. Both the crystal defects and the crowded growth environment caused by the “rich Ti, B area” are the fundamental reasons for the fragility and irregular shape of the TiB_2_.

## Figures and Tables

**Table 1:** Experiment parameters for Al–5Ti–B Master Alloy prepared by the halide salt route.

Temperature	Reaction Time							
800 °C	10 S	15 S	20 S	30 S	60 S	300 S	600 S	1800 S

**Table 2:** Experimental parameters for the partial Ti-sponge route and the Ti-sponge route.

Content of Ti	Reaction Time		
30% Ti-sponge route	15 S	300 S	1800 S
60% Ti-sponge route			
Ti-sponge route			
